# Cell-Free PURE System: Evolution and Achievements

**DOI:** 10.34133/2022/9847014

**Published:** 2022-08-30

**Authors:** Yi Cui, Xinjie Chen, Ze Wang, Yuan Lu

**Affiliations:** ^1^Key Laboratory of Industrial Biocatalysis, Ministry of Education, Department of Chemical Engineering, Tsinghua University, Beijing 100084, China; ^2^College of Life Sciences, Shenyang Normal University, Shenyang 110034, Liaoning, China

## Abstract

The cell-free protein synthesis (CFPS) system, as a technical core of synthetic biology, can simulate the transcription and translation process in an *in vitro* open environment without a complete living cell. It has been widely used in basic and applied research fields because of its advanced engineering features in flexibility and controllability. Compared to a typical crude extract-based CFPS system, due to defined and customizable components and lacking protein-degrading enzymes, the protein synthesis using recombinant elements (PURE) system draws great attention. This review first discusses the elemental composition of the PURE system. Then, the design and preparation of functional proteins for the PURE system, especially the critical ribosome, were examined. Furthermore, we trace the evolving development of the PURE system in versatile areas, including prototyping, synthesis of unnatural proteins, peptides and complex proteins, and biosensors. Finally, as a state-of-the-art engineering strategy, this review analyzes the opportunities and challenges faced by the PURE system in future scientific research and diverse applications.

## 1. Introduction

The purpose of cell-free synthetic biology is to understand, utilize, and expand the functions of natural biological systems without using the whole living cells. It breaks the boundary between nonliving chemicals and living bodies. Through purposeful design, transformation, and resynthesis of living systems, it even creates and endows “artificial life” with unnatural functions, thereby promoting innovation from mimicking life to recreating life [1–4]. The cell-free protein synthesis (CFPS) system is the core technology of cell-free synthesis biology [5], also known as *in vitro* protein transcription and translation technology.

Since the first successful construction of the CFPS system by Nirenberg and Matthaei [6], after nearly 70 years of research, people have found that the CFPS system has advantages in operation and application compared with the traditional cell system. In terms of operation, the CFPS system, as an *in vitro* life simulation system, avoids the tedious gene cloning and cell culture operations in the process of protein synthesis in the cell system. The CFPS system bypasses the cell wall, eliminates gene regulation, and can synthesize a large number of proteins in a short time. In addition to operability, the CFPS system has four advantages compared with the traditional cell system in application: safety, tolerance, storability, and fast response. First of all, CFPS does not involve live artificial transgenic cells, avoiding the risk of cell replication and transmission, so there is no biosafety problem [7]. Second, the CFPS system can operate in the presence of toxins [8], so it is tolerant to various chemical or biological agents. Third, through freeze-drying technology, the CFPS system can be stored stably for a long time [9]. Finally, its open environment avoids the obstacles of material transmembrane transport, so that the substance to be tested can be in direct contact with the reaction system, with shorter reaction time, higher sensitivity, and wider detection range. After years of development, modern CFPS systems can be divided into two types, including complex crude extract-based system and biochemically defined system, both of which provide a great degree of freedom for bioengineering.

Up to now, the crude extract-based system is the most broadly used complex CFPS system. It processes and breaks cells to remove insoluble substances and obtain necessary biochemical components for energy generation, transcription, and translation. Theoretically, almost all species can meet the basic needs of building a cell crude extract system [10]. A variety of CFPS systems with different cell crude extracts have been well developed [11]. For example, products that have been commercialized include NEBExpress Cell-free *E. coli* Protein Synthesis System (NEB), 1-Step Human In Vitro Protein Expression Kits (Thermo Scientific), and ALiCE® Mini Kit (Sigma). According to different sources, cell extracts can be divided into prokaryotic cell extracts and eukaryotic cell extracts [12, 13]. Among them, the CFPS system of *E. coli* is the most widely studied CFPS system at present. Although the CFPS system based on the cell crude extract already has the advantages that the traditional cell system cannot compare, we have to admit that it still has many shortcomings. There are still instabilities and uncertainties in the cell-free system using crude cell extracts, and there are great differences between different batches [14]. For example, a recent study found that although researchers in different laboratories use the same scheme to prepare the CFPS system, its variability is as high as 40.3% [15]. In addition to instability, it also has component uncertainty. For example, nucleases, ribonucleases, and proteases that cannot be removed can definitely have a negative impact on translation productivity [16, 17]. To solve these problems, researchers continuously optimized the CFPS system based on crude extract and, at the same time, vigorously developed a biochemically defined system.

A typical defined CFPS system, namely, protein synthesis using recombinant elements (PURE) system, was first developed by the Shimizu group in 2001 [18]. It is a CFPS system composed of purified components required for transcription and translation, in which all additives are completely known, and the concentration is controllable [19–21]. The PURE system has three apparent advantages over the crude extract-based system (Table 1). First, the composition of the PURE system is precise. It generally includes 36 purified proteins, tRNAs, ribosome, and necessary factors. There is no polluting protease in the PURE system, which makes the PURE system stable and deterministic. Second, the composition of each element in the PURE system can be adjusted according to different experimental needs to achieve the maximum protein expression, making the PURE system flexible [22–24]. Third, genetic code expansion or reprogramming is easier to be explored, and the manipulation of the translational machinery is easy in the PURE system [13, 25].

**Table 1:** Comparison of crude extract system and PURE system.

Features	Crude extract system	PURE system	References
Origin	1961	2001	[6, 18]
Composition	Crude extract (around 500-1000 complex proteins)+necessary factors	36 purified proteins+tRNAs+ribosome+necessary factors	[16, 17]
Preparation time	~4 days	>1 week	[26]
Chassis cell	Various prokaryotic cells or eukaryotic cells	*E. coli*; yeast	[13, 27–39]
mRNA or peptide degrading contaminants	Yes	No	[40]
Genetic code expansion or reprogramming	Hard to control due to complex extract	Easy to control due to defined components	[13, 25]
Manipulation of the translational machinery	Complex; less used	Easy; commonly used	[25, 41]
Cost	$0.3-0.5/*μ*L	$0.6-2/*μ*L	[42]
Applications	Versatile; high protein yield; high noise interference	Versatile; low protein yield; low noise interference	[25]

After nearly 20 years of research and development, the PURE system is considered to have significant advantages in basic biochemical research because of its transparent and controllable components [43]. This paper gives a comprehensive overview of the PURE system. The basic formulation of the PURE system is first introduced. Then, how to obtain the defined ingredients and key ribosome for assembling the PURE system was demonstrated. The latest research progress in the application fields of the PURE system was discussed, including prototyping, synthesizing, and biosensing. Furthermore, this review analyzes the opportunities and challenges that the PURE system may face. It is believed that the PURE system can be combined with more disciplines and technologies in the future to better apply to the research and development in various fields.

## 2. Construction of PURE Systems

### 2.1. Primary Components

**Figure 1:**
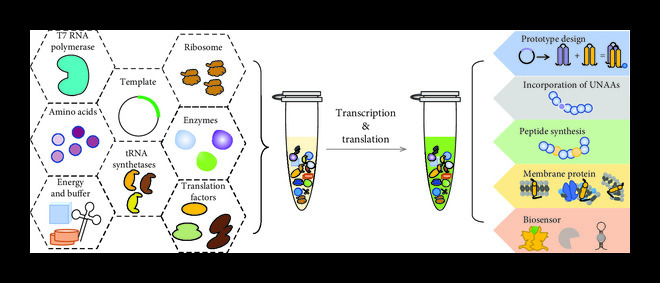
The components and applications of the PURE system.

The composition of the PURE system is defined, including purified proteins, ribosome, energy, and essential factors, as shown in Figure 1. The most critical part of the PURE system is the highly purified ribosome, followed by the purified proteins. These proteins include 10 *μ*g/mL T7 RNA polymerase for transcription, a full set of 20 tRNA synthetases for continuous tRNA aminoacylation (1900 U/mL AlaRS, 2500 U/mL ArgRS, 20 mg/mL AsnRS, 2500 U/mL AspRS, 630 U/mL CysRS, 1300 U/mL GlnRS, 1900 U/mL GluRS, 5000 U/mL GlyRS, 630 U/mL HisRS, 2500 U/mL IleRS, 3800 U/mL LeuRS, 3800 U/mL LysRS, 6300 U/mL MetRS, 1300 U/mL PheRS, 1300 U/mL ProRS, 1900 U/mL SerRS, 1300 U/mL ThrRS, 630 U/mL TrpRS, 630 U/mL TyrRS, and 3100 U/mL ValRS), translation factors for initiation, elongation, and termination (initiation factors, 2.7 *μ*M IF1, 0.4 *μ*M IF2, and 1.5 *μ*M IF3; elongation factors, 0.92 *μ*M EF-Tu, 0.66 *μ*M EF-Ts, and 0.26 *μ*M EF-G; release factors, 0.25 *μ*M RF1, 0.24 *μ*M RF2, and 0.17 *μ*M RF3; and ribosome recycling factor, 0.5 *μ*M RRF), and enzymes for energy cycle and regeneration (4 *μ*g/mL creatine kinase, 3 *μ*g/mL myokinase, 1.1 *μ*g/mL nucleoside-diphosphate kinase, 4500 U/mL methionyl-tRNA-formyltransferase, and 2 U/mL pyrophosphatase). Maintaining the PURE system’s reactions also requires energy and other component buffers, including 2 mM ATP, 2 mM GTP, 1 mM CTP, 1 mM UTP, 20 mM creatine phosphate, 50 mM HEPES–KOH PH 7.6, 100 mM potassium glutamate, 13 mM magnesium acetate, 2 mM spermidine, 1 mM DTT, 0.3 mM 20 amino acids, 10 mg/mL 10-formyl-5,6,7,8-tetrahydrofolic acid, and 56 U_A260_/mL tRNAs. These purified protein components are combined with ribosomes at a concentration of 1.2 *μ*M and the necessary templates to construct the PURE system [44].

In addition to homemade PURE systems, there are commercial PURE systems available, such as PUREfrex 2.0 (GeneFrontier), PURExpress (NEB) [45], and Magic PURE system (Creative Biolabs). Commercial PURE systems are efficient and easy to use but very expensive, around $0.6-2/microliter.

### 2.2. Ribosome Isolation

Ribosome is a highly complex intracellular macromolecule, mainly composed of rRNA and dozens of different r-proteins. The constituents of different species vary slightly. The ribosome of prokaryotes is composed of 65% rRNA and 35% r-protein [46], but the proportion of RNA and protein in eukaryotic ribosome is nearly identical [47]. The r-protein and rRNA are organized into the ribosomal large subunit (LSU) and the ribosomal small subunit (SSU) of ribosomes. Different sedimentation coefficients permit the categorization of ribosomes into the 70S and 80S types. The 70S ribosome mainly exists in prokaryotic cells. Its SSU unit is 30S, and its LSU unit is 50S. The 80S ribosome mainly exists in eukaryotic cells. The SSU unit is 40S, and the LSU unit is 60S. During protein synthesis, the LSU and SSU of ribosomes collaborate to transform mRNA into polypeptide chains.

The ribosome as the core translational machinery is the most critical component of the PURE system, but how to purify active ribosomes is the most challenging step [48]. The key factors for ribosome purification are cell quality and the purification strategy. The selection of strains used to purify ribosomes and the timing of cell collection impacts final ribosome quality. Compared with common strains, strains lacking RNase I, such as MRE600, A19, JE28, and Q13, are the preferred *E. coli* strains to purify ribosomes [49], reducing ribosomal RNA degradation to a certain extent. MRE600 (ATCC 29417) lacks RNase I [50]. A19 (CGSC 5997) has six mutations of rna-19, gdhA2, his-95, relA1, spoT1, and metB1 [51]. In JE28, hexahistidine affinity tag has been inserted at the C-terminus of the ribosomal protein L12 [52]. Q13 is a mutant of A19 [53]. Strains used to obtain ribosomes should grow rapidly in a rich medium at the optimum temperature and be harvested in the early and medium logarithmic stages before growth and translation begin to slow down.

**Figure 2:**
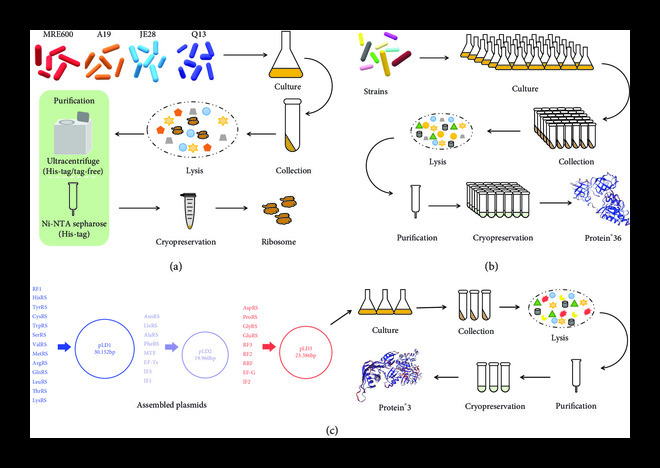
Purification of ribosome and functional proteins for the PURE system. (a) Different strains used for isolating ribosomes without or with His-tag. (b) Method of purifying functional proteins individually. (c) Purifying functional proteins based on three assemble plasmids.

The ribosome purification can be divided into two ways according to whether they have His-tag or not, as shown in Figure 2(a). One way is introducing His-tag into three ribosome subunit genes, and the ribosome can be directly and simply purified using Ni-NTA Sepharose. It has been proved that the purified ribosome labeled with His-tag in 50s or 30S ribosomal proteins has the same activity as 70S ribosome purified using traditional sucrose gradient centrifugation [54]. After the development and improvement of this method [52], more laboratories can prepare purified ribosomes, because it only needs standard laboratory equipment to operate, which can further facilitate the preparation of the PURE system. The other way is traditionally purifying ribosomes by hydrophobic interaction and sucrose gradient centrifugation. Although this method can be applied to the purification of both tag-free ribosome and His-tag labeled ribosome, it requires that the laboratory is equipped to use a liquid chromatography system and ultracentrifuge [55]. In some studies, it was found that the activity of His-tag ribosome was significantly lower than that of unlabeled variants, and in some cases, this low yield may be acceptable [26]. Although the purification strategy of ribosomes has a significant impact on the activity of the PURE system, RNA degradation and protease pollution in the environment are inevitable in the process of ribosome purification. How to avoid such degradation and pollution is still a challenging research direction for future efforts.

### 2.3. Functional Proteins

Obtaining high-quality purified functional proteins except the ribosome is another key part of preparing the PURE system. After years of ongoing experimental studies, there are two main methods for purifying proteins in the PURE system. One is to purify each protein one by one (Figure 2(b)) and then add and mix each protein according to the amount required after all purification. Another purification method is combined purification from microbial consortia or bacterial artificial chromosomes. For example, a total of 30 translation factors with His-tag were encoded into three high-copy expression plasmids, and only three times of batch purification was further performed [56], as shown in Figure 2(c). These two methods have their own advantages and disadvantages. Although the method of individual purification takes a long time and is troublesome to operate, it can control the concentration of each protein added to the PURE system. However, while the combined purification method can greatly shorten the time required for protein purification and successfully establish the PURE system, it is unable to control the concentration of each protein. What needs to be emphasized is that, in the combined method, there are still six proteins that cannot be encoded on plasmids, which need to be purified separately, including T7 RNA polymerase, EF-Tu, myokinase, nucleoside-diphosphate kinase, pyrophosphatase, and creatine phosphate. In particular, EF-Tu needs to be purified separately, because it requires a higher level than any other translation factor.

### 2.4. Different Chassis

In theory, any organism can be the chassis for constructing the CFPS system, but due to still unclear translation mechanism and cumbersome preparation process, only two chassis cells are developed, including *E. coli* and yeast. In 2001, researchers first used *E. coli* to establish the PURE system, because it has clear genomic information and the characteristics of simple culture conditions, fast growth, low culture cost, simple cell lysis method, and high protein yield [57]. Up to now, *E. coli* is still the most commonly used model strain of the PURE system. As people become more and more familiar with engineering the PURE system, researchers are no longer satisfied with the prokaryote research of *E. coli* but turn their attention to how to use eukaryotes to establish the PURE system and carry out relevant research and application.

Yeast *Saccharomyces cerevisiae*, as one of the most typical eukaryotes, first attracted the attention of researchers. Although cap-dependent initiation is the most complex translation stage in yeast, it requires at least 12 initiation factors and tRNA aminoacylation process, which has always been a difficulty in yeast translation. In a recent study, researchers developed CrPV IGR IRES (intergenic region internal ribosome entry site sequence from cricket paralysis virus)-containing mRNA [58], which enables yeast 80S ribosome to be started without promoter tRNA or any eukaryotic translation promoter. Long peptides can be synthesized *in vitro* by combining CrPV IGR IRES with a recombinant translation system composed of components from yeast and *E. coli* (yeast: elongation factors eEF1A and eEF2, tRNA mix, and recycling factor Rli1; *E. coli*: elongation factors eEF3, termination factors eRF1 and eRF3, and recycling factors Hbs1 and Dom34). The emergence of this yeast-derived PURE system proved that eukaryotic translation factor eIF5A could not only synthesize long peptides [59] but also alleviate polyproline-mediated ribosomal stall [60] by eIF5A and its hypusine modification.

The development of PURE systems with different chassis cells will be potentially useful for various applications. Taking the PURE system of yeast as an example, it is applicable to the design of drugs that act on eukaryotic ribosome (such as antibiotics against fungi) and the study of corresponding structures and functions. Combined with a ribosome- or mRNA-display system, this system can be used as a powerful *in vitro* screening system for new functional proteins, such as antibacterial peptides with cytotoxicity. It is believed that with the emergence of PURE systems developed by more different chassis cells, the PURE systems will have more application fields and can play a broader role through the combination with other systems or disciplines.

### 2.5. Optimization Tactics

The emergence of the PURE system has further expanded the application scope of the CFPS system [55], but as a protein synthesis technology, it still has the problems of high reagent cost, small reaction scale, and low yield of recombinant protein. Based on this research background, two optimization tactics have been adopted, including regulating system components and molecular crowding. Using the same reagent dosage to produce more proteins can make the PURE system relatively cheap. By adjusting the concentrations of ribosome, translation factors (especially EF-Tu), release factors, and initiation factors, the protein yield of the PURE system can be increased by up to five times. However, the concentrations of these elements are not the higher, the better. When the PURE system reaches the maximum protein output, if the concentration of each element continues to increase, the productivity of the PURE system would be inhibited [40, 48, 61–63].

In addition to adjusting the component concentrations, adding bovine serum albumin (BSA) to the PURE system or aggregating the functional proteins of the PURE system around nanoparticles can increase the environmental molecular crowding, which in turn can increase the protein synthesis in the PURE system. By cross-linking the PURE system enzymes onto quantum dots, the formed nanoaggregates can improve the synthesis of the fluorescent protein and phosphotriesterase with a 12-fold increase [64]. This may be achieved by bringing components closer together or optimizing crowding effects that have not yet been defined.

Accordingly, adjusting the component concentrations and environmental molecular crowding can improve protein synthesis. However, it must be said that the concentrations of some elements in the PURE system cannot be adjusted even if necessary. For example, magnesium ion affects the ribosome function, but it is difficult to control its concentration because it can be chelated by negatively charged molecules, such as NTPs, creatine phosphate, and pyrophosphate. Therefore, developing more effective and convenient tactics to improve protein synthesis is still an urgent challenge that PURE systems have to face now and even in the future.

## 3. Cutting-Edge Applications of PURE System

The PURE system has realized the rapid expression of recombinant protein. Its flexibility, controllability, and convenient operation make it used in many application fields. This section discusses the latest research progress of the PURE system in different fields, including prototype design, incorporation of unnatural amino acids, peptide synthesis, complex protein synthesis, and biosensors.

### 3.1. Prototype Design

**Figure 3:**
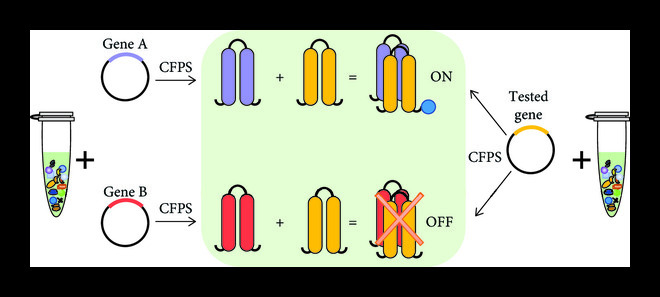
Application of PURE system in prototype design.

In prototyping applications, the idea is to use CFPS systems to test and optimize biosynthetic pathways before implementation and amplification in living cells. The PURE system is based on purified components and therefore is flexible and adaptable to different reaction settings. It can synthesize many proteins difficult to express in cells [18]. As shown in Figure 3, the adaptability of the PURE system is very useful in the prototype design of candidate drugs without protein purification, which can not only synthesize various therapeutic molecules but also readily explore the effect of drugs on specific pathways [65]. For example, one study synthesized nonribosomal peptide natural products (NRPs) indigoidine and rhabdopeptide through the PURE system [66]. Apart from strong adaptability, the PURE system also features short response time. Shortening the time from the beginning to the end of the reaction is another important advantage of the PURE system in prototype design. Moreover, the PURE system can simulate the communication process of cells based on gene networks combined with microfluidic technology. For example, the PURE reaction generates *α*-haemolysin after light activation, so as to create a communication channel between individual droplets in a programmable way. The LacY transporter generated *in situ* by the PURE reaction realizes the active transport of signal molecules, to realize the programmed specific communication between two droplets [67]. Prototype design is a proof-of-concept stage that must be screened and verified before industrial applications. No matter the applications of drug test or gene circuit test, the strong adaptability and short response time of the PURE system can make it the best choice of prototype design.

### 3.2. Unnatural Amino Acid Incorporation

**Figure 4:**
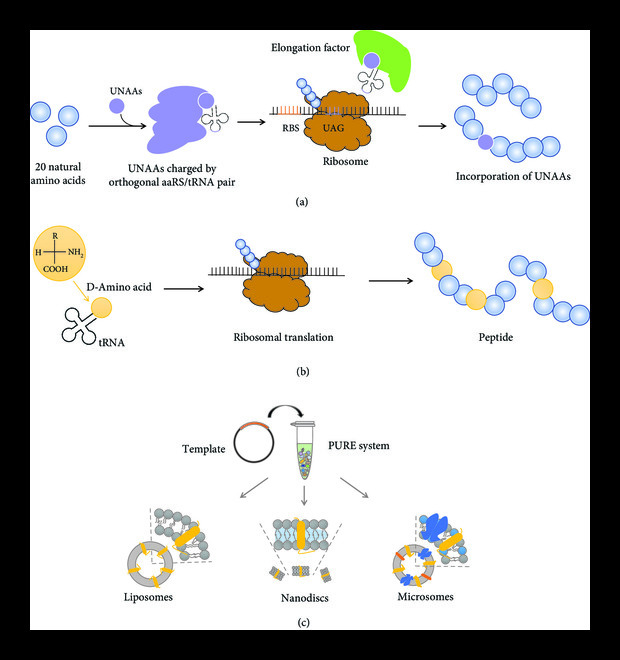
Biosynthesis applications of PURE system: (a) application of PURE system in the unnatural amino acids; (b) application of PURE system in peptide synthesis; (c) application of PURE system in membrane protein synthesis.

The embedding of unnatural amino acids (UNAAs) [68] with new side chain groups can give proteins new chemical properties, forming novel protein structures and functions [69]. It opens the door to new protein engineering, provides a new way for biological research, biotherapy, and synthetic biology, and has become a key emerging application in frontier fields [70]. Based on orthogonal translation systems (OTSs), the UNAA incorporation methods include stop codon suppression, frameshift suppression, codon redistribution, and unnatural base pairs [13]. The defined chemical properties of the PURE system can help to customize tRNA and aminoacyl-tRNA synthetases selectively according to application needs (Figure 4(a)). The latest research on combining OTSs with the PURE system demonstrated that selective omission of RF1 release factor into the PURE system could enhance the inhibition of biorthogonal tRNA and allow the incorporation of UNAAs at multiple sites [71, 72]. The use of mutant EF-Tu in the PURE system can improve the binding efficiency of UNAAs with a large number of side chains [73]. Through the expansion of genetic codes [74] and the systematic evolution of tRNA/aminoacyl-tRNA synthetase homologs, the PURE system can incorporate more than 50 different UNAAs into proteins and can generate new chemical or functional properties in proteins by adding fluorescent markers or reaction groups [75, 76]. Twenty natural amino acids (NAAs) can also produce natural proteins with a variety of structures and functions; however, the current natural protein cannot meet the research needs of protein engineering and protein drug production. The PURE system is suitable for the application of unnatural amino acid incorporation and synthesis of complex unnatural proteins, which will make the PURE system a powerful unnatural protein engineering platform to meet the growing demand for new types of applications.

### 3.3. Peptide Synthesis

With the emergence and development of Flexizyme (flexible tRNA acylation ribozyme), the PURE system has become a robust platform for producing nonstandard peptides (Figure 4(b)). The combination of the PURE system and Flexizyme can produce various cyclized peptides, including backbone cyclization, backbone-side chain cyclization, and bicyclic and tricyclic peptides [77–80]. These peptides not only contain more than 300 different UNAAs, such as N-methyl and D-amino acids, but also have the potential as drugs.

Moreover, by combining the PURE system with Flexizyme tRNA aminoacylation technology and mRNA display, a powerful *in vitro* peptide selection platform was built [81, 82], which can generate a macrocyclic peptide library with a diversity of more than 1000. The formation of this library could contribute to the development of reagents, affinity ligands, and drugs [83]. For example, studies have found that HIP-8 can selectively recognize active hepatocyte growth factors, which may contribute to potential cancer diagnosis and treatment [84]. The ub4ix can specifically bind to the K48-linked ubiquitin chains and protect it from degradation by ubiquitinase. The formed macrocyclic peptide can enter cells and form a therapeutic intervention effect by inhibiting cell growth or inducing programmed death [85]. The PURE system only contains low levels of nuclease, ribonuclease, and protease activities [86, 87], which not only allows the use of linear DNA or mRNA as the translation template of effective peptides but also has little negative impact on protein translation. These characteristics make the PURE system the first choice as the research platform for peptide synthesis.

### 3.4. Complex Protein

Membrane proteins play an important role in biological activities such as cell proliferation and differentiation, energy conversion, signal transduction, and material transport and account for more than 60% of the current drug target proteins [88]. The PURE system has the characteristics of controllable reaction conditions and high toxicity tolerance, which enables it to stably synthesize complicated membrane proteins *in vitro*. The key support component for membrane protein synthesis is the biomembrane. Membrane simulants (such as liposomes, nanodiscs, and microsomes) can be added to the open CFPS reaction to assist the membrane protein synthesis and assembly [89–91], as shown in Figure 4(c). Using the PURE system, multiple membrane protein subunits can be prepared in parallel without cumbersome cDNA cloning or gene synthesis steps [92]. The PURE system can also express some difficult, complex proteins that cannot be expressed in cells or traditional CFPS systems [93]. For example, this system has been successful in producing active dihydrofolic acid reductase (DHFR) [94]. In addition, the researchers have successfully produced insulin analogs with the same structure and affinity for receptors as those produced by yeast. This suggests that the PURE system is also suitable for expressing soluble molecules with higher-order features and multiple disulfide bridges [95]. The PURE system can stabilize the synthesis of membrane proteins and other complex proteins, so that it could be applied to not only the basic research of biological macromolecules but also the research and development of drugs, contributing to the pharmaceutical field.

### 3.5. Biosensor Diagnostics

**Figure 5:**
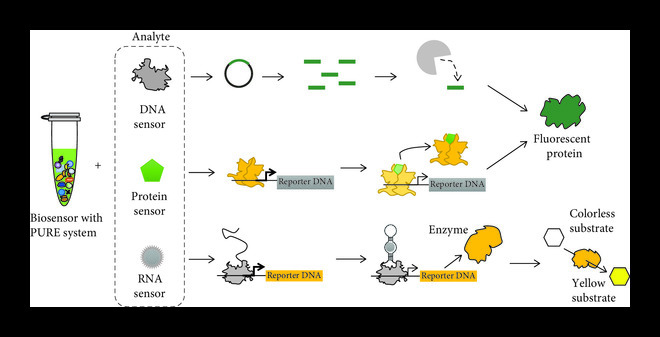
Application of PURE system in biosensors.

The development of the PURE system combined with freeze-drying technology or microfluidic technology can realize small volume biosensing, contributing to the application of on-site biosensors, such as point-of-care diagnosis and water quality testing [96], as shown in Figure 5. A new cell-free biosensor platform, RNA output sensors activated by ligand induction (ROSALIND), has been successfully developed with the PURE system. ROSALIND consists of a highly processive phage RNA polymerase, aTFs, and engineered DNA transcription templates, which together produce visible outputs upon exposure to specific ligands. Using RNA-level outputs enables the observation of signals within minutes and eliminates the requirement for sophisticated and resource-intensive protein translation to identify responses. The key advantage of ROSALIND in transcriptional RNA output is designing RNA circuits to solve the well-known shortcomings of transcription factors without protein engineering. ROSALIND can detect various compounds and elements in water, which can help fill the gap in existing water quality monitoring technology and meet the needs of communities and individuals to detect water quality quickly and cheaply [97].

The PURE system can be used not only for environmental monitoring but also for disease diagnosis. A low-cost colorimetric analysis method has been established, which can detect norovirus and Zika virus from clinical samples by combining cell-free RNA sensing technology with the PURE system and constant temperature amplification [98]. The biosensor system can generate a signal proportional to the concentration of the analyte through the interaction between the sensor and the target analyte [8, 99]. The defined composition and good sensitivity of the PURE system can help develop biosensors with small volume and convenience. For example, the combination of the PURE system and colorimetric reporter enzyme can generate eye-readable biosensor readings, so that no additional equipment is required for further interpretation [100]. This indicates that the PURE system has potential biosensing diagnostics in multiple application scenarios.

## 4. Conclusion and Prospects

As a powerful platform for cell-free synthetic biology technology, the PURE system developed significantly in the past five to ten years. Due to its openness, controllability, efficiency, flexibility, and many other advantages, it has been widely used, from the basic research of prototype design to the application research of biosynthesis and biosensing. However, the development of the PURE system still faces many challenges, especially in industrial applications.

Cost and scale-up are two major concerns for PURE systems in industrial manufacturing. The application expansion and development of the PURE system are limited by the cost of reagents. The high cost comes mainly from cumbersome preparation steps, low biosynthesis yield, and low system stability. Only on the basis of the cost issue being solved, the scale-up issue can be carried out further. To reduce the cost and scale up the reaction, we can seek solutions from the following aspects: simplifying the component preparation process, exploring molecular chaperones to improve protein synthesis, realizing effective energy regeneration, achieving good system stability and quality control, and designing suitable bioreactors.

As engineering problems are solved, the application potential of the PURE system needs to be further exploited. Through the fusion of artificial intelligence and computer design, natural biomolecules or networks with improved or novel functions can be prototyped and screened. With the advancement of material science, synthetic biology, physics discipline, chemistry discipline, and electronic engineering, their seamless integration with the open and flexible PURE system will open up a new world of applications in biocatalysis and human health.

## Abbreviations

CFPS: Cell-free protein synthesis
PURE: The protein synthesis using recombinant elements
LSU: Ribosomal large subunit
SSU: Ribosomal small subunit
*E. coli*: *Escherichia coli*
CrPV IGR IRES: Intergenic region internal ribosome entry site sequence from cricket paralysis virus
BSA: Bovine serum albumin
NRPs: Nonribosomal peptide natural products
UNAAs: Unnatural amino acids
OTSs: Orthogonal translation systems
NAAs: Natural amino acids
Flexizyme: Flexible tRNA acylation ribozyme
DHFR: Dihydrofolic acid reductase
ROSALIND: RNA output sensors activated by ligand induction.
